# Estimating malaria incidence from routine health facility-based surveillance data in Uganda

**DOI:** 10.1186/s12936-020-03514-z

**Published:** 2020-12-02

**Authors:** Adrienne Epstein, Jane Frances Namuganga, Emmanuel Victor Kamya, Joaniter I. Nankabirwa, Samir Bhatt, Isabel Rodriguez-Barraquer, Sarah G. Staedke, Moses R. Kamya, Grant Dorsey, Bryan Greenhouse

**Affiliations:** 1 Department of Medicine, University of California, San Francisco, 550 16th Street, San Francisco, CA 94158 USA; 2 Infectious Diseases Research Collaboration, Kampala, Uganda; 3 Department of Internal Medicine, Makerere University College of Health Sciences, Kampala, Uganda; 4 Department of Infectious Disease Epidemiology, St Marys Hospital, Imperial College, London, UK; 5 London School of Hygiene and Tropical Medicine, London, UK; 6 Chan Zuckerberg Biohub, San Francisco, CA USA

**Keywords:** Surveillance, Health management information system, Uganda, Incidence, Health facility, Malaria

## Abstract

**Background:**

Accurate measures of malaria incidence are essential to track progress and target high-risk populations. While health management information system (HMIS) data provide counts of malaria cases, quantifying the denominator for incidence using these data is challenging because catchment areas and care-seeking behaviours are not well defined. This study’s aim was to estimate malaria incidence using HMIS data by adjusting the population denominator accounting for travel time to the health facility.

**Methods:**

Outpatient data from two public health facilities in Uganda (Kihihi and Nagongera) over a 3-year period (2011–2014) were used to model the relationship between travel time from patient village of residence (available for each individual) to the facility and the relative probability of attendance using Poisson generalized additive models. Outputs from the model were used to generate a weighted population denominator for each health facility and estimate malaria incidence. Among children aged 6 months to 11 years, monthly HMIS-derived incidence estimates, with and without population denominators weighted by probability of attendance, were compared with gold standard measures of malaria incidence measured in prospective cohorts.

**Results:**

A total of 48,898 outpatient visits were recorded across the two sites over the study period. HMIS incidence correlated with cohort incidence over time at both study sites (correlation in Kihihi = 0.64, p < 0.001; correlation in Nagongera = 0.34, p = 0.045). HMIS incidence measures with denominators unweighted by probability of attendance underestimated cohort incidence aggregated over the 3 years in Kihihi (0.5 cases per person-year (PPY) *vs* 1.7 cases PPY) and Nagongera (0.3 cases PPY *vs* 3.0 cases PPY). HMIS incidence measures with denominators weighted by probability of attendance were closer to cohort incidence, but remained underestimates (1.1 cases PPY in Kihihi and 1.4 cases PPY in Nagongera).

**Conclusions:**

Although malaria incidence measured using HMIS underestimated incidence measured in cohorts, even when adjusting for probability of attendance, HMIS surveillance data are a promising and scalable source for tracking relative changes in malaria incidence over time, particularly when the population denominator can be estimated by incorporating information on village of residence.

## Background

Malaria surveillance is widely recognized as an essential intervention to target regions and populations at high risk, accurately measure changes in disease burden, and evaluate the impact of interventions [1]. In many high burden settings, surveillance is conducted through passive case detection at health facilities as part of the routine health management information system (HMIS). There are several strengths in conducting HMIS-based surveillance: data provide direct measures of morbidity, are collected continuously over time, and cover a broad geographic range [2]. However, these data are often hindered by reporting delays and gaps, poor data quality, health-seeking behaviour, and lack of laboratory-confirmed diagnostics [3, 4]. For this reason, measures of malaria morbidity assessed with HMIS data tend to largely underestimate true burden [1, 3, 5–7].

An additional challenge with the utility of HMIS surveillance data is in translating case counts into meaningful metrics of malaria burden. A common HMIS-derived metric is the test positivity rate (TPR), defined as the proportion of individuals who test positive for malaria per 100 individuals tested. The TPR has several inherent limitations: it is prone to bias due to the incidence of non-malarial illnesses, has a non-linear relationship with malaria incidence, and cannot be translated into absolute estimates of incidence [8–11]. The most useful metric of malaria morbidity is malaria incidence, defined as the number of cases of malaria per unit time divided by the size of the population at risk [8, 12]. The major challenge of translating HMIS data into accurate measures of malaria incidence is quantifying the denominator, because catchment areas around health facilities are not well defined. Previous efforts to quantify this denominator have relied on representative cross-sectional surveys with information on household care-seeking [13, 14], an additional source of information that is costly to collect and requires population-level representativeness.

The aim of this study was to estimate malaria incidence over time, without the need for independent survey data on care seeking, using enhanced HMIS data. This study leveraged high quality, individual-level HMIS surveillance data, including information on village of residence for patients presenting to two Uganda Malaria Surveillance Programme (UMSP) Malaria Reference Centres (MRCs) over 3 years from 2011 to 2014. The relationship between travel time and outpatient attendance was modelled to generate a weighted population denominator for each MRC and estimate incidence over time. HMIS-derived incidence estimates where then compared to gold standard measures of malaria incidence measured prospectively in cohort studies conducted in sub-counties surrounding MRCs.

## Methods

### Study sites

This analysis used data from health facility-based malaria surveillance systems in two Ugandan sub-counties: Kihihi sub-county, Kanungu district and Nagongera sub-county, Tororo district. Both sub-counties are rural; at the time of the study, Kihihi exhibited moderate transmission intensity (annual entomological inoculation rate [aEIR] 2011–2013 = 32.0) and Nagongera high transmission intensity (aEIR = 310) [15]. Both regions experience two annual peaks in malaria burden following the rainy seasons.

From 2013–2014, the government of Uganda carried out a universal distribution of free long-lasting insecticide-treated nets (LLINs) with the goal of achieving one net per two people in each household. Nagongera sub-county received nets in November 2013 and Kihihi sub-county received nets in June 2014.

### Health facility-based data

Enhanced malaria surveillance was established via the UMSP MRCs in 2006, as previously described [16]. UMSP conducts sentinel surveillance in 70 level III and IV public outpatient facilities in Uganda, including Kihihi Health Centre IV and Nagongera Health Centre IV. At each MRC, individual-level outpatient department records are entered into an electronic MS Access (Microsoft Corporation, Redmond, WA) database for all individuals presenting to the outpatient departments of the health facilities using a standardized format. Data collected includes patient demographics (age, gender, and village of residence), results of laboratory tests (rapid diagnostic test or microscopy), diagnoses given, and treatments prescribed. UMSP provides laboratory support and quality control training to ensure high quality diagnostic testing. Data are sent to the UMSP data centre and cleaned before transfer to Stata (Stata Corp, College Station, TX) for analysis. This analysis uses 3 years of health-facility based surveillance data from the two MRCs (September 2011-August 2014). These months were selected given the low level of missingness (< 30%) for village of residence. This analysis was restricted to patients aged 6 months through 10 years to make them comparable to cohort data described below.

### Cohort data

Dynamic cohort studies were conducted in children aged 6 months through 10 years from 100 households randomly selected from the two study sub-counties, as previously reported [15]. In summary, eligible children from selected households were followed from August 2011 through June 2017. At enrollment, parents/guardians provided written informed consent and received an LLIN. Cohort participants received free medical care at designated study clinics located at the same MRCs where UMSP data were being collected; parents/guardians were encouraged to bring their children to the clinic any time they were ill. Children who presented with a fever (tympanic temperature ≥ 38.0 °C) or history of fever in the previous 24 h had a thick blood smear performed. If the blood smear was positive by microscopy, the child was diagnosed with malaria and provided treatment. Episodes of uncomplicated malaria were treated with artemether-lumefantrine; complicated or recurrent malaria occurring within 14 days of prior therapy was treated with quinine.

### Measures

#### Malaria suspected

Health-facility based surveillance recorded all outpatients as “malaria suspected” or “malaria not suspected.” Malaria suspected was defined as patients who a) underwent a laboratory test for malaria (microscopy or rapid diagnostic test); or b) were given a clinical diagnosis of malaria in the absence of laboratory testing. Any record that did not meet these criteria was considered “malaria not suspected.”

#### Malaria cases

At MRCs, malaria cases were defined as patients with laboratory-confirmed malaria diagnoses (by microscopy or rapid diagnostic test).

#### Gold standard incidence

Malaria incidence measured through dynamic cohorts was considered the gold standard. Incidence was defined as the number of new episodes of malaria divided by the total person time observed. New episodes of malaria were defined as any episode of malaria not preceeded by another episode in the prior 14 days. A secondary definition using a parasite threshold of 2000 parasites/μL was also applied as a sensitivity analysis.

### Statistical analysis

#### Travel time estimation

Villages located within Kihihi and Nagongera sub-counties were mapped during cross-sectional enumeration surveys conducted in 2009–2010 [15]. These village shapefiles were linked to unique identifiers of villages found in the UMSP database. Villages of residence for all outpatients living within the MRC subcounty were identified and mapped.

Travel times were calculated using Malaria Atlas Project’s friction surface 2015 raster file obtained through Google Earth Engine, available at 1-km resolution [17]. The authors of this friction surface combined datasets on roads, railways, water bodies, slope and elevation, landcover, and borders to calculate a nominal overall speed of travel across each pixel, in units of minutes of travel time per metre. Travel times represent Uganda-specific mean travel times associated with the road types in the pixel, or, in pixels where no roads are present, walking times. The malariaAtlas R package was used to calculate the mean travel time from each outpatient’s village to the MRC of interest, in addition to the travel times to all nearest level III and IV health facilities [18]. Travel times were defined as the minimum travel time between two points.

#### Care-seeking model

Observations were restricted to those residing in villages whose nearest level III or IV health facility was the MRC of interest, assuming that individuals attend their nearest health facility. These villages were defined as the MRC’s “catchment area” [13]. Since not all individuals seek care when ill, and this care-seeking behaviour is driven in part by distance to the health facility, this analysis sought to account for this distance-specific care-seeking rather than using the raw population of the catchment area as a denominator for incidence. The probability of seeking care at the MRC was expected to decay as function of travel time to the facility. Relative village-level care-seeking probabilities were modelled and estimated as a function of travel time to the facility from each village within the catchment area. These probabilities were then used to down-weight village populations when estimating incidence. For example, if care-seeking from a particular village was estimated to be 80%, the population seeking care from that village was estimated to be 80% of the total population.

The care-seeking model was restricted to outpatients for whom malaria was not suspected. This group was used because their probability of attendance should be minimally biased by heterogeneity in malaria incidence across villages. Because this population represents a range of diagnoses, spatial bias is expected to be minimal. By using this population to model care-seeking, this analysis assumed that differences in care-seeking for outpatients not suspected of having malaria over space was driven solely by travel time to the health facility.

For each MRC, non-linear Poisson generalized additive models (GAMs) were specified to estimate the relationship between mean travel time from village *i* to the MRC and the count of outpatients not suspected of having malaria who visited the MRC from village *i* from September 2011–2014. GAMs are a class of generalized linear models that allow for the relationship between the outcome and predictor to be estimated using smooth functions of the predictor variables [19]. A non-parametric smooth function was applied to the travel time predictor, as the relationship between travel time to the facility and attendance was hypothesized to be non-linear. An offset for the logged population from village *i* derived from the High Resolution Settlement Layer [20] was included. To calculate relative village-level probabilities of attendance, predicted counts were estimated using the model described above holding the village population size constant. These counts were rescaled to relative probabilities by dividing the predictions by the predicted count in the village where the MRC is located. Calculating the relative probabilities in this way assumes that individuals living in the same village as the MRC have a probability of seeking care of 1.

In order to evaluate the sensitivity of these findings to the aforementioned assumptions, models were re-specified restricting outpatients to the top 5 diagnoses (including malaria) to determine whether the relationship between travel time and attendance differed across indications. In addition, stratified analyses were performed based on age category (6 months to < 5 years, 5 years to < 11 years) and gender. Models were also specified using straight-line distance from the centroid of the village of residence to the MRC as predictor and compared to results using travel time as predictor.

#### Incidence estimation

HMIS data were used to estimate malaria incidence in two ways. First, incidence was estimated by dividing malaria cases over the catchment area denominator (including all villages for which the MRC is the closest health facility) without down-weighting for travel time, hereafter called unweighted catchment incidence. Second, malaria incidence was estimated by dividing malaria cases by a weighted denominator using the weights described above to adjust village-level populations, hereafter called weighted catchment incidence. All populations were set to grow at a fixed rate each month based on the World Bank’s estimate of population growth during the study window (0.29% monthly) [21]. Both of these HMIS-derived measures were compared to metrics of gold standard (cohort) incidence by generating plots over time, calculating measures of pair-wise correlation by month, and comparing aggregated estimates of malaria incidence over the three year study window. This method assumes that relative treatment-seeking behaviour for non-malarial illness is the same as for malaria.

## Results

**Fig. 1:**
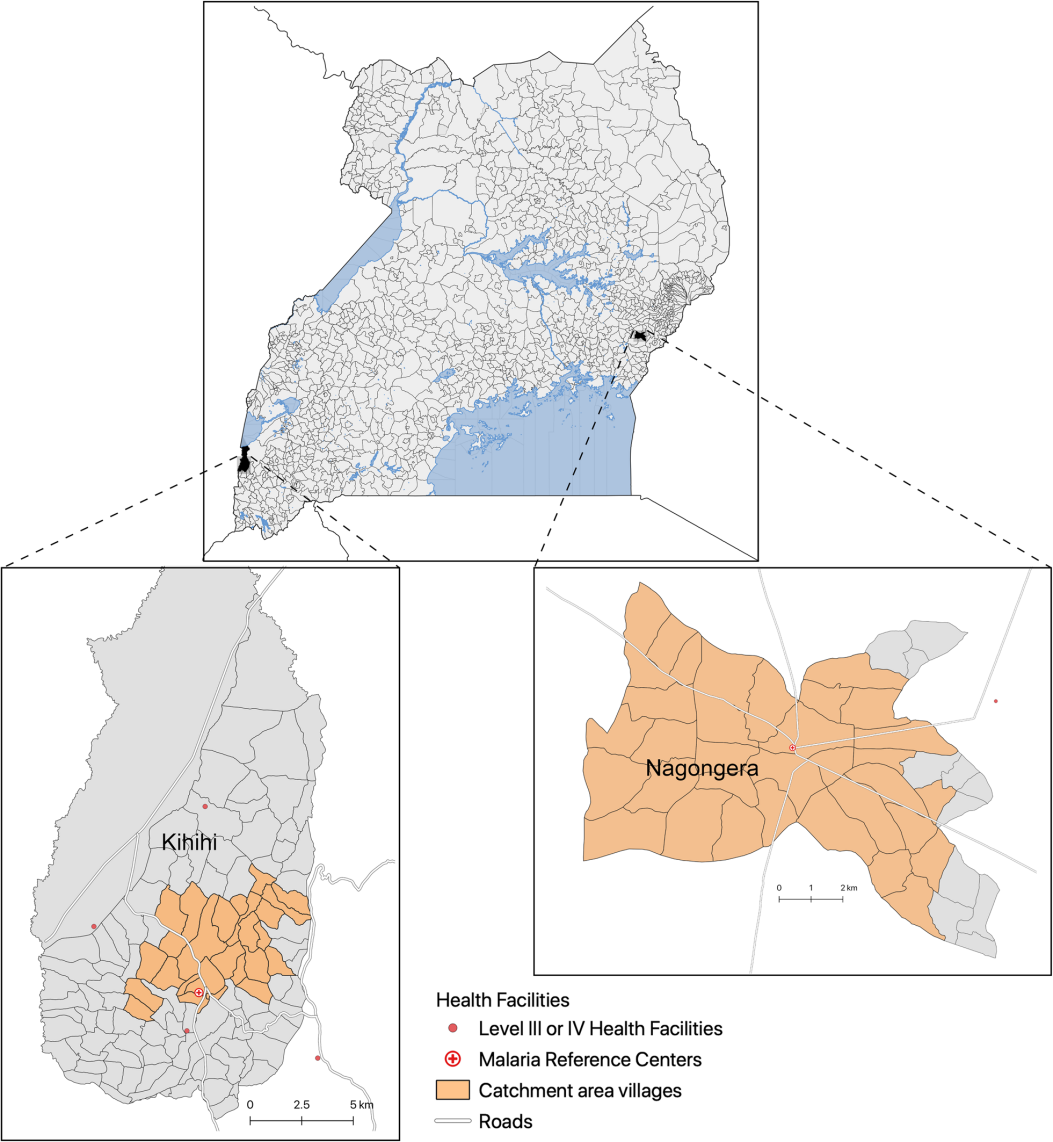
Map Malaria Reference Centers and their catchment areas in Kihihi and Nagongera sub-counties

Of the 118 villages mapped in Kihihi sub-county, 30 villages were included in the catchment area, totaling a population of 15,155 (Fig. 1). Mean village-level travel times to the MRC ranged from 0 to 40 min (mean 13 min). In Nagongera sub-county, 30 of 45 villages were included in the catchment area, totaling a population of 32,226. Travel times to the MRC ranged from 1 to 21 min (mean 10 min).

Health facility-based surveillance involved a total of 48,898 visits among children aged 6 months to 11 years over the 3-year observation period (Table 1). A total of 46.1% and 49.7% of these visits occurred among patients residing within the catchment areas of Kihihi and Nagongera, respectively. The proportion of outpatient visits from within the catchment areas suspected of having malaria was 88.9% and 88.7% in the two sub-counties, and over 98% of these individuals underwent laboratory testing. The TPR within the catchment area was 50.0% in Kihihi and 43.8% in Nagongera. For the cohort studies, a total of 686 children were observed over 1,628 person-years over the 3-year observation period. A total of 3,778 episodes of malaria were diagnosed, with an average malaria incidence of 1.7 and 3.0 cases per person-year (PPY) at risk in Kihihi and Nagongera, respectively.

**Table 1:** Summary statistics from health facility-based and cohort surveillance studies; September 2011–August 2014

Data source	Metric	Study site	
		Kihihi	Nagongera
Malaria Reference Centres	Outpatient visits for children aged 6 months–< 11 years	20,742	28,156
	Outpatient visits for children aged 6 months–< 11 years from catchment area (percent of total)	9,555 (46.1%)	13,985 (49.7%)
	Malaria suspected from catchment area (percent of total from catchment area)	8,497 (88.9%)	12,401 (88.7%)
	Diagnostic test performed (percent of malaria suspected from catchment area)	8,493 (99.9%)	12,238 (98.7%)
	Tested positive for malaria in catchment area (percent of tested from catchment area)	4,247 (50.0%)	5,358 (43.8%)
Cohort Studies	Number of children observed	353	333
	Person-years of observation	848	780
	Number of episodes of malaria	1,474	2,304
	Incidence of malaria (new episodes per person-year)	1.7	3.0

**Fig. 2:**
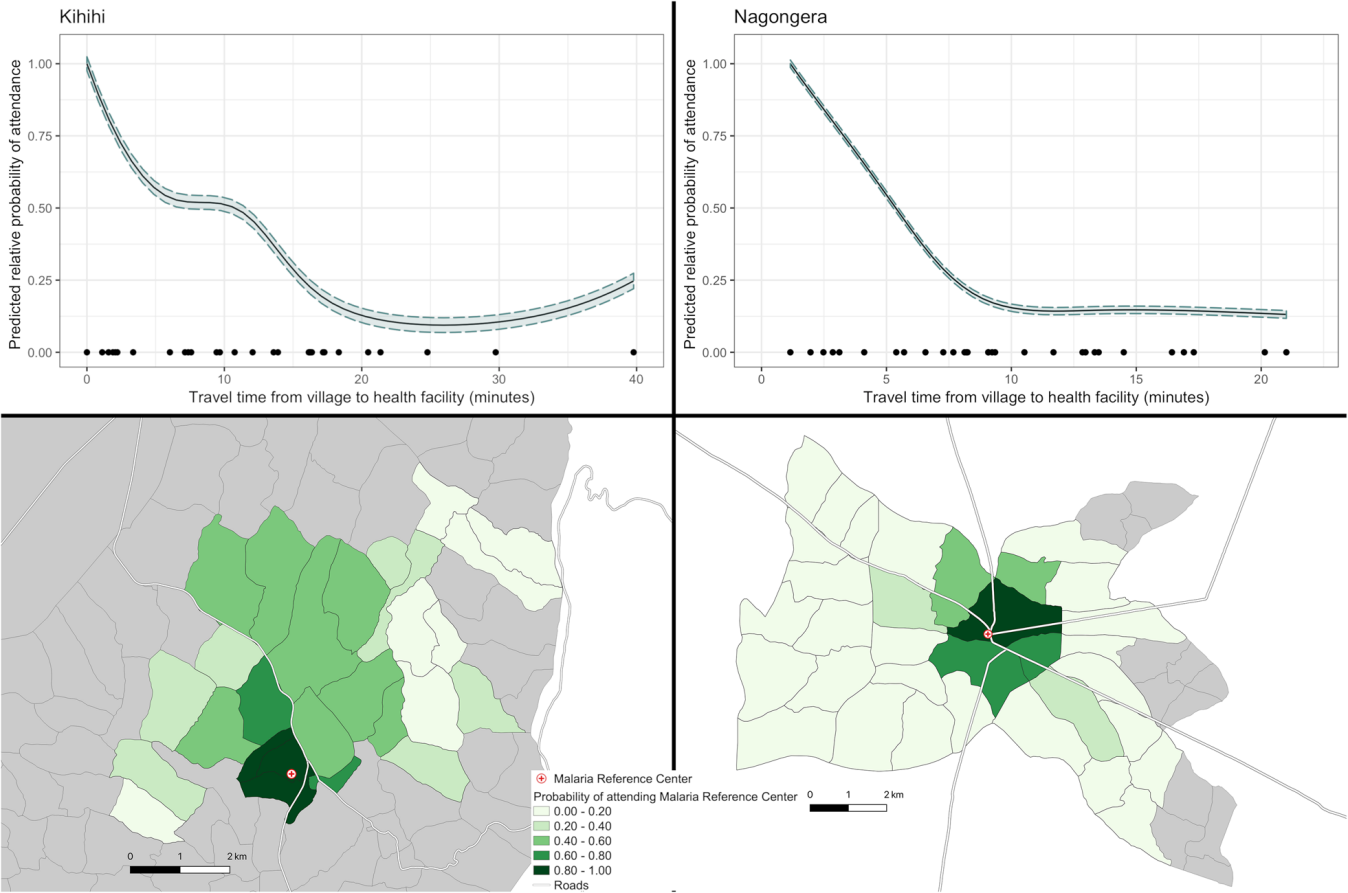
Modelled relationship between travel time to the health facility and probability of attending the health facility (top) and map of village-level probabilities of attendance (bottom). The modelled relationships (top) were derived from non-linear Poisson generalized additive models; 95% confidence intervals are shaded in green and points on bottom represent the distribution of villages in the catchment area. Greyed out villages (bottom) represent villages in the subcounty not included in the catchment area

The relationship between travel time and the predicted probability of attendance is presented in Fig. 2, and as expected decreased with increasing travel time in both sites (see Additional file 1 for the data included in this analysis). In Kihihi, the probability of attendance dropped steadily, plateaued at approximately 10 min, then continued to drop, with a slight increase at the furthest village included in the catchment area. In Nagongera, the probability of attendance dropped steadily until approximately 10 min travel time, then flattened at close to 10%. The shape of these curves was substantively similar to curves resulting from models using straight-line distance as predictor (Additional file 2). The relationship between travel time and attendance was consistent across age groups and sexes (Additional files 3 and 4). In Nagongera, these relationships were also consistent when stratifying by diagnosis. However, in Kihihi, the relationship between travel time and probability of attendance differed among those diagnosed with malaria, cough or cold, diarrhoea, and GI disorders, with a lower probability of attendance in the village where the health facility was located compared to villages with travel times around 10 min (Additional file 5). Of note, the village where the MRC is located in Kihihi is urban and has a documented lower level of malaria transmission than surrounding villages [22].

**Fig. 3:**
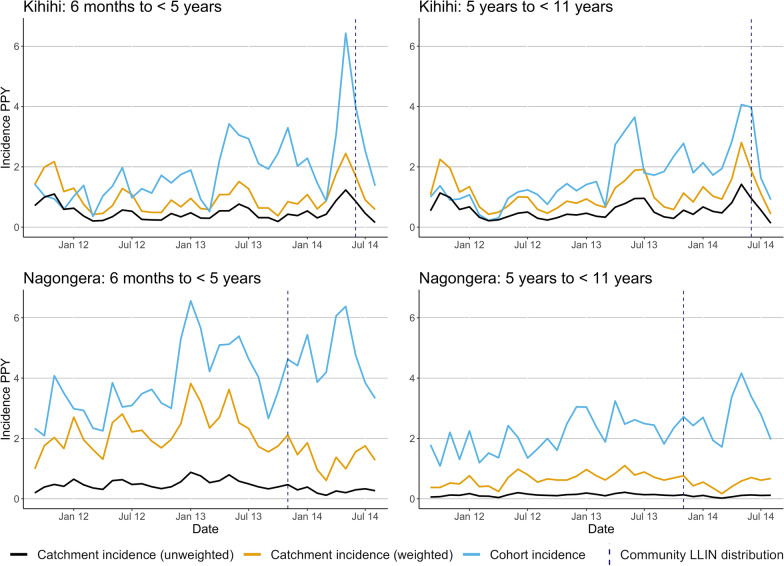
Incidence of malaria over the 3-year observation period measured in cohorts and using health facility-based surveillance

The three incidence measures (weighted catchment incidence, unweighted catchment incidence, and cohort incidence) are plotted over time by age group in Fig. 3. In most months across age groups and sites, both the weighted and unweighted catchment measures followed the same trajectory of cohort incidence. Weighted catchment incidence underestimated cohort incidence (with the exception of the first several months of observation in Kihihi), but less so than the unweighted measure. In Nagongera, weighted catchment incidence followed cohort incidence until the community-level LLIN distribution, when they diverged. When a parasite density of 2000 parasites/μL was applied to incident cohort cases the results were similar, but cohort incidence fell closer to weighted catchment incidence (Additional file 6). The pairwise correlation between cohort and catchment incidence (both weighted and unweighted) was higher in Kihihi (corr = 0.64, p < 0.001) than Nagongera (corr = 0.34, p = 0.045). However, when restricting to the period of time prior to the universal LLIN distribution, the correlation in Nagongera was higher (corr = 0.72, p < 0.001).

Incidence estimates by age group and metric (cohort, weighted, and unweighted) aggregated over the 3-year observation period are presented in Table 2. These reflect the findings plotted in Fig. 3, with the weighted incidence metric falling between cohort and unweighted incidence.

**Table 2:** Malaria incidence PPY measured at surveillance sites from September 2011–August 2014

	Kihihi		Nagongera		Nagongera (pre-November 2013 LLIN distribution)	
	6 months to < 5 years	5 years to < 11 years	6 months to < 5 years	5 years to < 11 years	6 months to < 5 years	5 years to < 11 years
Cohort incidence	1.79	1.70	3.95	2.29	3.72	2.11
HMIS Weighted incidence	1.01	1.11	1.99	0.63	2.22	0.67
HMIS Unweighted incidence	0.50	0.55	0.43	0.12	0.50	0.13

**Fig. 4:**
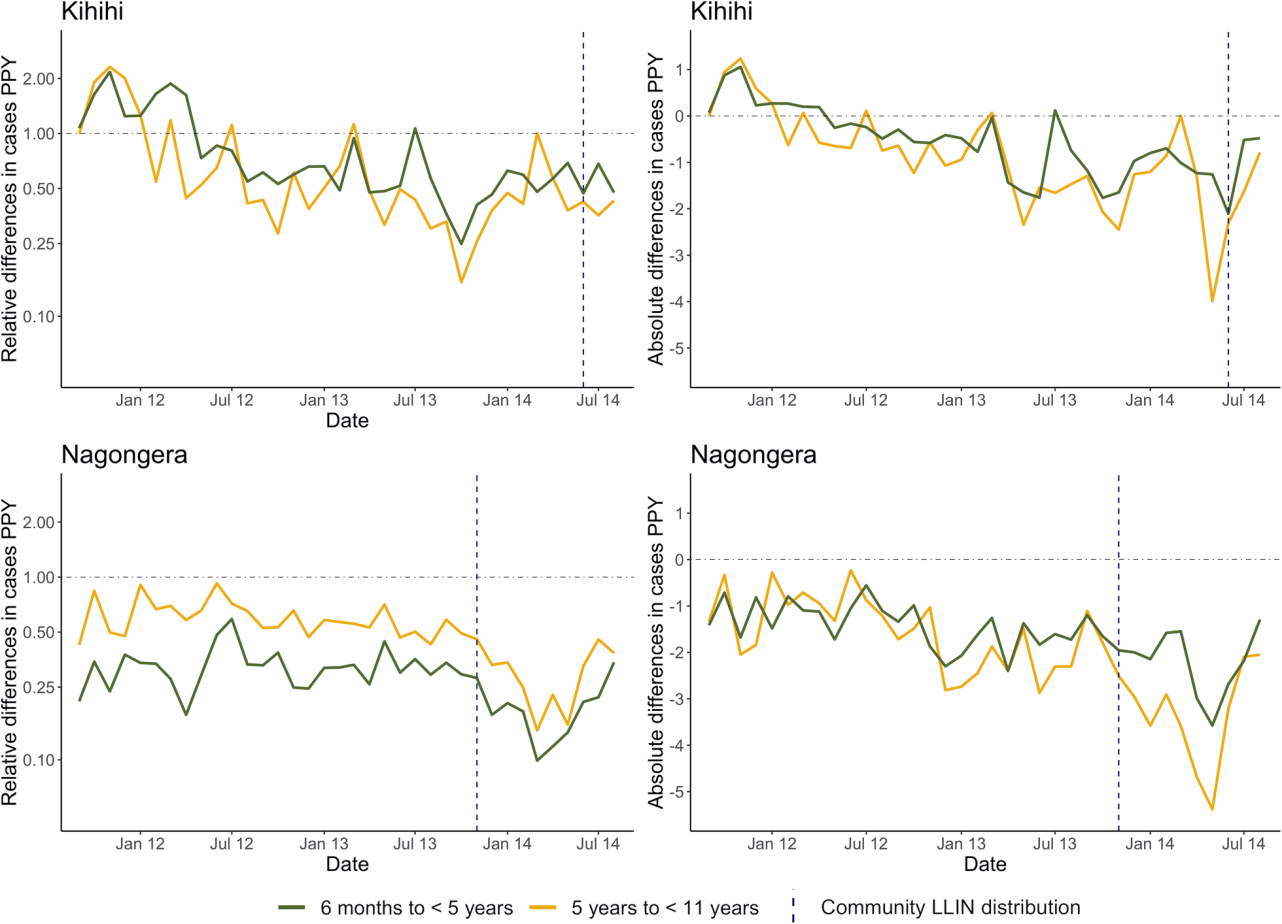
Absolute and relative differences between weighted and cohort incidence

To best understand bias in incidence estimates derived from enhanced HMIS data, weighted catchment incidence was compared to cohort incidence for each month, stratified by age (Fig. 4). In Kihihi, health facility-based incidence initially overestimated cohort incidence (approximately twofold or 1 episode of malaria PPY), then underestimated incidence (approximately 50% or 2 episodes of malaria PPY, Fig. 4). This trend in overestimation followed by underestimation was consistent across age groups. In Nagongera, weighted catchment incidence consistently underestimated cohort incidence, particularly after community level LLIN distribution: during the final year of observation. Unlike Kihihi, different degrees of bias in estimation by age group were observed, with relative incidence in younger children consistently underestimated to a larger degree than older children. In absolute terms, however, the differences were very similar between age groups until after community level LLIN distribution where incidence was underestimated to a larger degree in older children.

## Discussion

This study used routinely collected HMIS data to estimate malaria incidence longitudinally and validated these estimates by comparing them to gold standard measures in moderate and high burden settings. Findings suggest that temporal changes in HMIS-based measures correlated reasonably well with a gold standard measure of incidence over time. Weighted estimates, which leveraged information on village of residence and travel time to the health facility to account for differences in care-seeking behaviour, fell much closer to the gold standard incidence than unweighted estimates that incorrectly assumed all individuals in the assigned catchment areas had a uniform probability of attendance. However, even using weighted estimates, HMIS data produced estimates of malaria incidence that were consistently lower than estimates from cohort studies, suggesting that not all episodes of malaria were being captured through the HMIS system. Nevertheless, these findings contribute to a broader literature indicating that HMIS data, particularly when analysed accounting for care-seeking behaviour, have potential to provide a relatively inexpensive data source to estimate key metrics of malaria burden across space and over time [12, 15, 23].

Findings from this study indicate that as travel time to the facility increased, the probability of health facility attendance fell precipitously. This was especially true in Nagongera, where the probability of attendance decreased by 50% as the travel time increased by onlt 5 min. One potential explanation is that the friction surface’s resolution was too crude (1 km × 1 km) for village-level estimates. However, the steepness of these curves were also found when straight-line distance was used as a predictor. Another possibility is that there may be private health facilities and pharmacies within the area competing for care-seeking; the location of these facilities were not considered when estimating the catchment area.

Estimating malaria incidence from HMIS data has surveillance and programmatic benefits. A common measure derived from HMIS data is the TPR, which is often used as a proxy measure for measuring temporal trends in malaria incidence [12] and assessing the impact of control interventions [24, 25]. However, the TPR is not informative about absolute case counts and, therefore, cannot be used for planning purposes (for example, when determining counts of anti-malarial medications to send to facilities) nor for estimating cases averted by control interventions. This is because the TPR correlates poorly with malaria incidence, especially in particularly low and high transmission settings, and does not capture differences between facilities [10]. Incidence, alternatively, is an absolute measure of burden in the population; using HMIS data to measure malaria incidence longitudinally, therefore, would allow trends in the absolute burden of disease to be tracked over time and across space.

There are several potential reasons why HMIS-based measures consistently underestimated cohort incidence even after down-weighting the population denominator. First, the assumption that care-seeking is 100% in the village where the facility is located may be incorrect; if care-seeking is lower, true incidence is underestimated. Second, there are key differences between the populations that participated in the cohort studies and the broader population throughout the sub-county. The cohorts represented a unique situation where barriers to care-seeking were removed through travel reimbursement, minimal waiting time and no hidden costs; therefore, health facility attendance was essentially universal. The underestimation of the weighted HMIS measure may therefore be explained by differences in care-seeking behaviour other than travel time, such as financial and time burdens or care-seeking at different facilities, such as lower level public facilities or private facilities. In Nagongera, this underestimation was more pronounced in older age groups. This may be due to differential care-seeking behaviours for caregivers of older children; this phenomenon (lower rates of care-seeking among caregivers of older children compared to children under 5) has been previously reported in Ethiopia and Malawi [26, 27].

One potential reason is that older children have greater immunity to clinical malaria and therefore a higher threshold for seeking care. Another potential explanation is that older children in this high transmission setting commonly have asymptomatic parasitaemia. In the cohort setting, these children may be more likely to seek care if they had fever (for any reason) and, in the presence of asymptomatic parasitaemia, would be classified as clinical/symptomatic malaria. Thus, the cohort incidence may in fact overestimate incidence of malaria episodes in older children in high burden settings, which is consistent with the results from applying a parasite threshold of 2000 parasites/μL to the definition of cohort incidence. It is unclear why these findings—the observed differential underestimation of incidence by age—were not echoed in Kihihi, though this could be related to the lower transmission in Kihihi compared with Nagongera.

There was a divergence of HMIS and cohort incidence following the 2013 universal LLIN distribution in Nagongera. The 2013 distribution was the first universal LLIN distribution in Uganda. Evidence suggests that LLIN ownership and use was quite low before the distribution; the 2011 Uganda Demographic and Health Survey found that only 27% of households had at least one LLIN per two people [28]. Participants in the cohort were given LLINs upon enrollment and, therefore, may have already experienced the individual and household-level benefits of LLIN utilization on malaria incidence; this divergence may be due to the broader population receiving LLINs later in the observation period and the likelihood of community-level benefits of LLIN use. Previous work concluded that there was no significant change in malaria incidence among cohort participants following community-level LLIN distribution in Nagongera [29]. These results suggest that the impact on community incidence may have been larger than previously indicated based on the cohort data alone.

This study contributes to the literature by proposing a novel method to more accurately estimate malaria incidence from HMIS data using improved estimates of the population denominator. Previous work to estimate care-seeking probabilities to apply to incidence denominators has relied on representative, cross-sectional surveys that ask individuals about their care-seeking behaviour [13, 14, 30–32]. These surveys are costly and are not conducted regularly, and the questions are often non-specific in that they do not ask respondents which health facility they attended. This study instead leveraged continuously available outpatient information on geographic location of residence, information that is part of the standard outpatient registers at Ugandan health facilities. Estimating catchments using this information has utility beyond measuring incidence of malaria, such as assessing access to care in low-resource, high-burden settings [33], and assessing seasonal changes in health-seeking behaviour. If not already collected, health facility systems should consider adding geographic information to their routine data collection. In countries where these data are collected, Ministries of Health may consider an investment in training and support for health workers to ensure data completeness and accuracy; in recent years, UMSP has emphasized data completeness for geographic variables and brought missingness down to below 5% across all 70 sites. These data could then be linked to geocoded information on administrative units, data which are increasingly publicly available, allowing for georeferenced information on patients’ origins.

This study has several limitations. First, the gold standard used in this study—malaria incidence measured in cohorts of children 6 months to 11 years of age—may not represent the true malaria incidence in the underlying community. Care-seeking patterns in the cohorts were in the setting of a research study and may not reflect real world behaviours. Second, absolute care-seeking probabilities were not possible to estimate with these data. This is because cross-sectional survey data on care-seeking behaviours in the villages around the MRCs are not available. Thus, the estimates of the denominator for incidence represent an improved upper bound compared to estimates without weighting, and the estimates of malaria incidence represent an improved lower bound. The inability to generate absolute probabilities poses challenges with comparing incidence between health facilities because care-seeking behaviours may differ across sites. However, treatment for malaria is free in Uganda and there is some evidence that care-seeking is quite high: the 2018–2019 Malaria Indicator Survey found that 87% of caregivers sought treatment for children with fever in the 2 weeks preceding the survey [34]; this figure may be higher in villages that are closest to the health facility. Thus, the assumption that care-seeking is close to 100% in the village where the health facility is located may be plausible. Finally, the High Resolution Settlement Layer, which combines satellite, census, and Facebook data to generate high resolution population estimates [20] for the population denominator undoubtedly contain uncertainty.

## Conclusions

This study underscores the potential for HMIS data to estimate key metrics of malaria burden. Although cases captured at the health facility will likely continue to underestimate true burden, health facility metrics with estimation of population denominators accounting for care seeking may still allow for measurements of changes in burden over time. In practice, estimating catchment area denominators using down-weighting may be best applied in sentinel surveillance sites across high burden countries, due to the required methodological and time investments. Alternatively, instead of using a model to estimate care-seeking, health systems could aim to measure where people reside and catchment areas could be defined including patients living immediately around the health facility where care-seeking can be assumed to be essentially universal, notably in Uganda where public health care is free. This would require a modest investment in time and training of health professionals to include geographic information in the collection of patient demographics. With this information, HMIS data can be used to generate quality measures of malaria incidence that are relatively inexpensive, an essential tool for countries around the globe as they aim to achieve targets towards control and elimination.

## Supplementary information

**Additional file 1: **Data for care-seeking model.

**Additional file 2: **Modelled relationship between distance between village centroid and the health facility and probability of attending the health facility.

**Additional file 3: **Predicted probabilities and 95% confidence intervals of attending the health facility among patients not suspected of having malaria stratified by age.

**Additional file 4: **Predicted probabilities and 95% confidence intervals of attending the health facility among patients not suspected of having malaria stratified by gender.

**Additional file 5: **Predicted probabilities and 95% confidence intervals of attending the health facility stratified by the top 5 most common diagnoses.

**Additional file 6: **Incidence of malaria over the 3-year observation period measured in cohorts with additional parasite threshold definition and using health facility-based surveillance.

## Data Availability

The datasets used and/or analysed for this study are available from the corresponding author upon request. The PRISM cohort datasets are available publicly from ClinEpiDB at https://clinepidb.org/.

## Abbreviations

HMIS: Health management information system
TPR: Test positivity rate
aEIR: Annual entomological inoculation rate
LLIN: Long-lasting insecticide-treated nets
UMSP: Uganda Malaria Surveillance Programme
MRC: Malaria Reference Centre
GAM: Generalized additive model
PPY: Per person-year
