# Supplementary material for: Estimating malaria incidence from routine health facility-based surveillance data in Uganda
Source: Malar J. 2020 Dec 2;19:445. doi: 10.1186/s12936-020-03514-z (PMC7709253; doi:10.1186/s12936-020-03514-z)

Additional File 2. Modeled relationship between distance between village centroid and the health facility and probability of attending the health facility

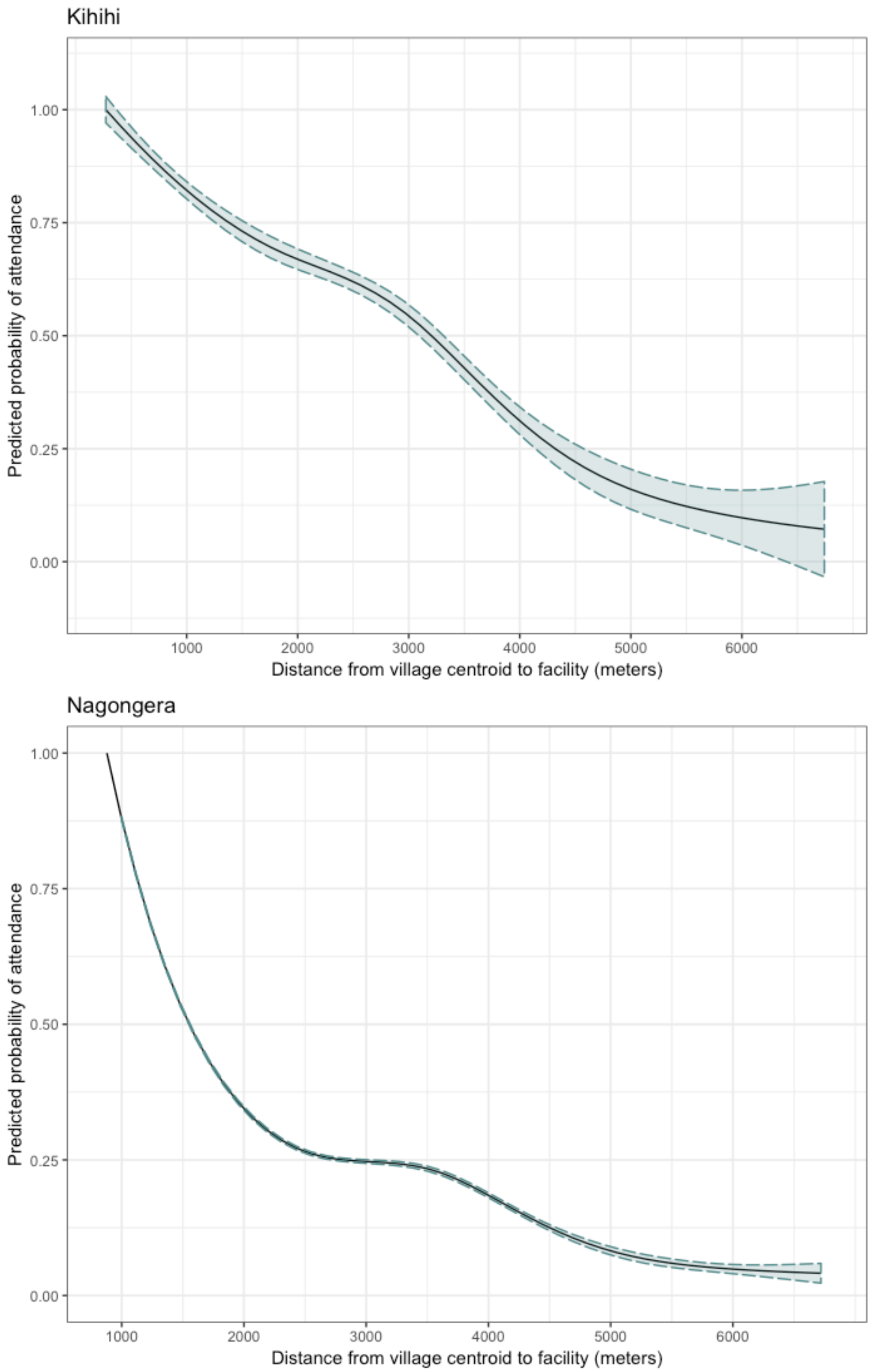

Supplement: Supplementary file 2 — Additional file 2: Modelled relationship between distance between village centroid and the health facility and probability of attending the health facility. [file 12936_2020_3514_MOESM2_ESM.pdf]
