# Supplementary material for: Estimating malaria incidence from routine health facility-based surveillance data in Uganda
Source: Malar J. 2020 Dec 2;19:445. doi: 10.1186/s12936-020-03514-z (PMC7709253; doi:10.1186/s12936-020-03514-z)

Additional File 4. Predicted probabilities and 95% confidence intervals of attending the health facility among patients not suspected of having malaria stratified by sex.

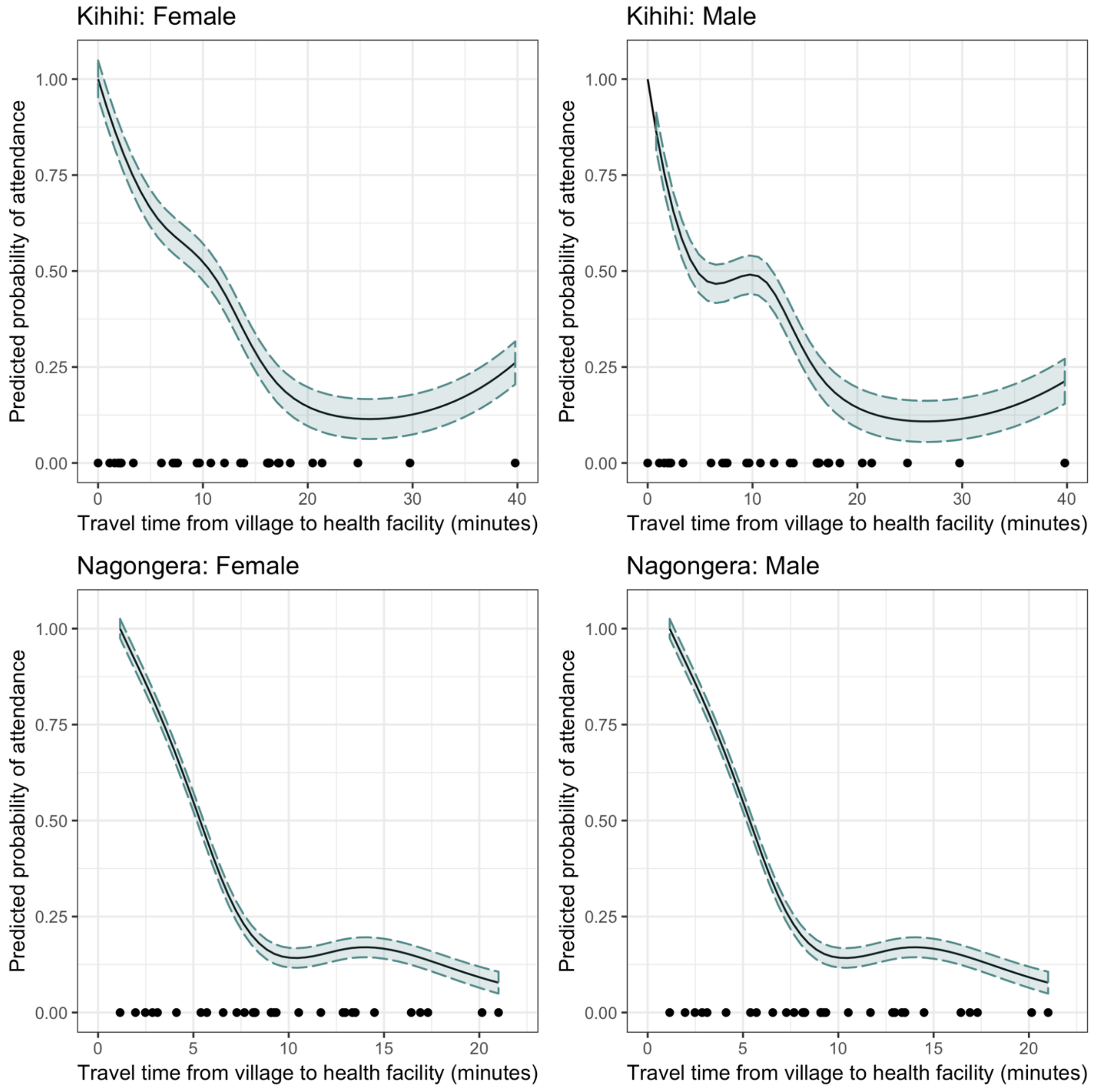

Supplement: Supplementary file 4 — Additional file 4: Predicted probabilities and 95% confidence intervals of attending the health facility among patients not suspected of having malaria stratified by gender. [file 12936_2020_3514_MOESM4_ESM.pdf]
