# Supplementary material for: Estimating malaria incidence from routine health facility-based surveillance data in Uganda
Source: Malar J. 2020 Dec 2;19:445. doi: 10.1186/s12936-020-03514-z (PMC7709253; doi:10.1186/s12936-020-03514-z)

Additional File 5. Predicted probabilities and 95% confidence intervals of attending the health facility stratified by the top 5 most common diagnoses.

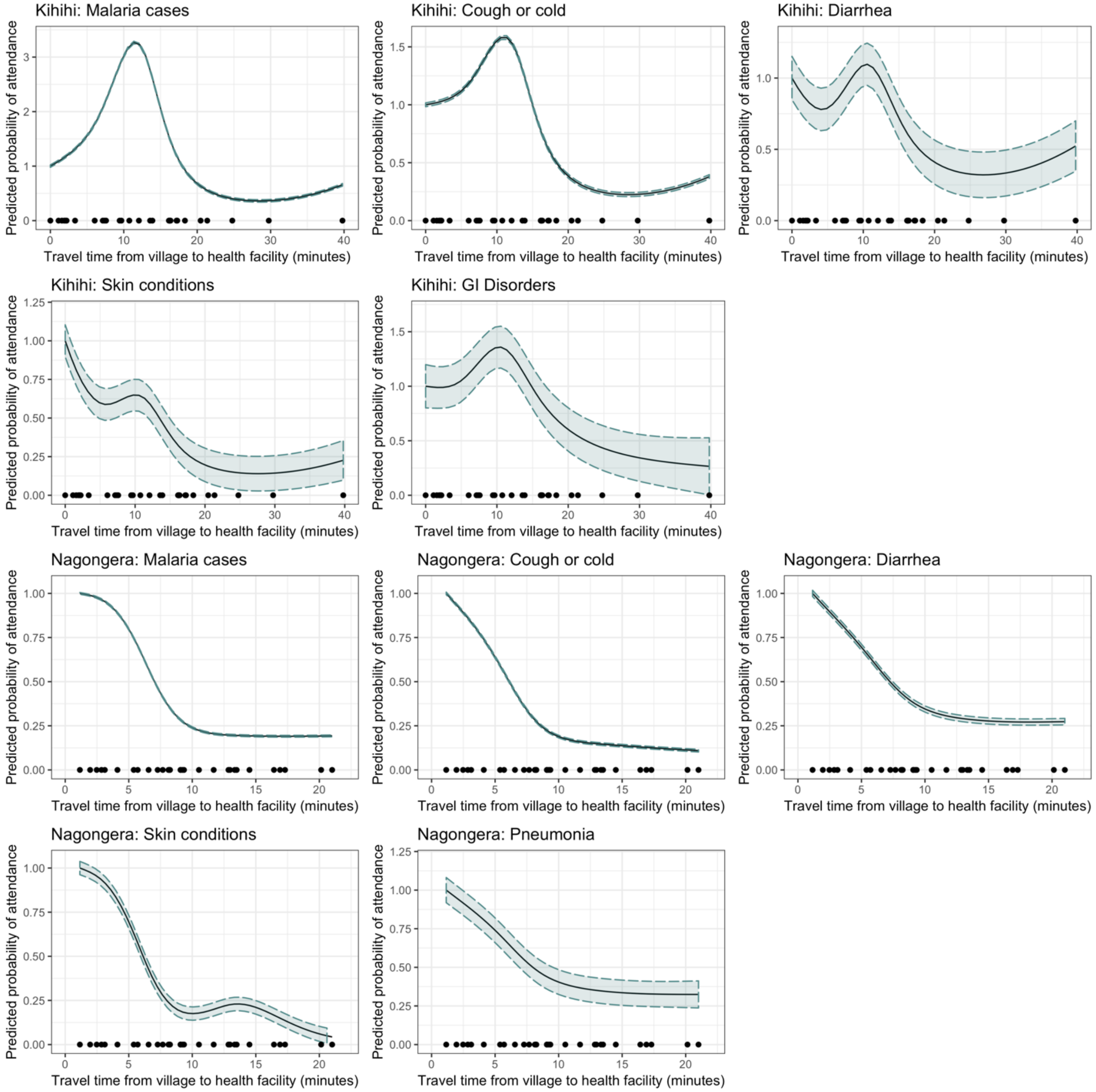

Supplement: Supplementary file 5 — Additional file 5: Predicted probabilities and 95% confidence intervals of attending the health facility stratified by the top 5 most common diagnoses. [file 12936_2020_3514_MOESM5_ESM.pdf]
