# Supplementary material for: Estimating malaria incidence from routine health facility-based surveillance data in Uganda
Source: Malar J. 2020 Dec 2;19:445. doi: 10.1186/s12936-020-03514-z (PMC7709253; doi:10.1186/s12936-020-03514-z)

Additional File 6. Incidence of malaria over the 3-year observation period measured in cohorts with additional parasite threshold definition and using health facility-based surveillance.

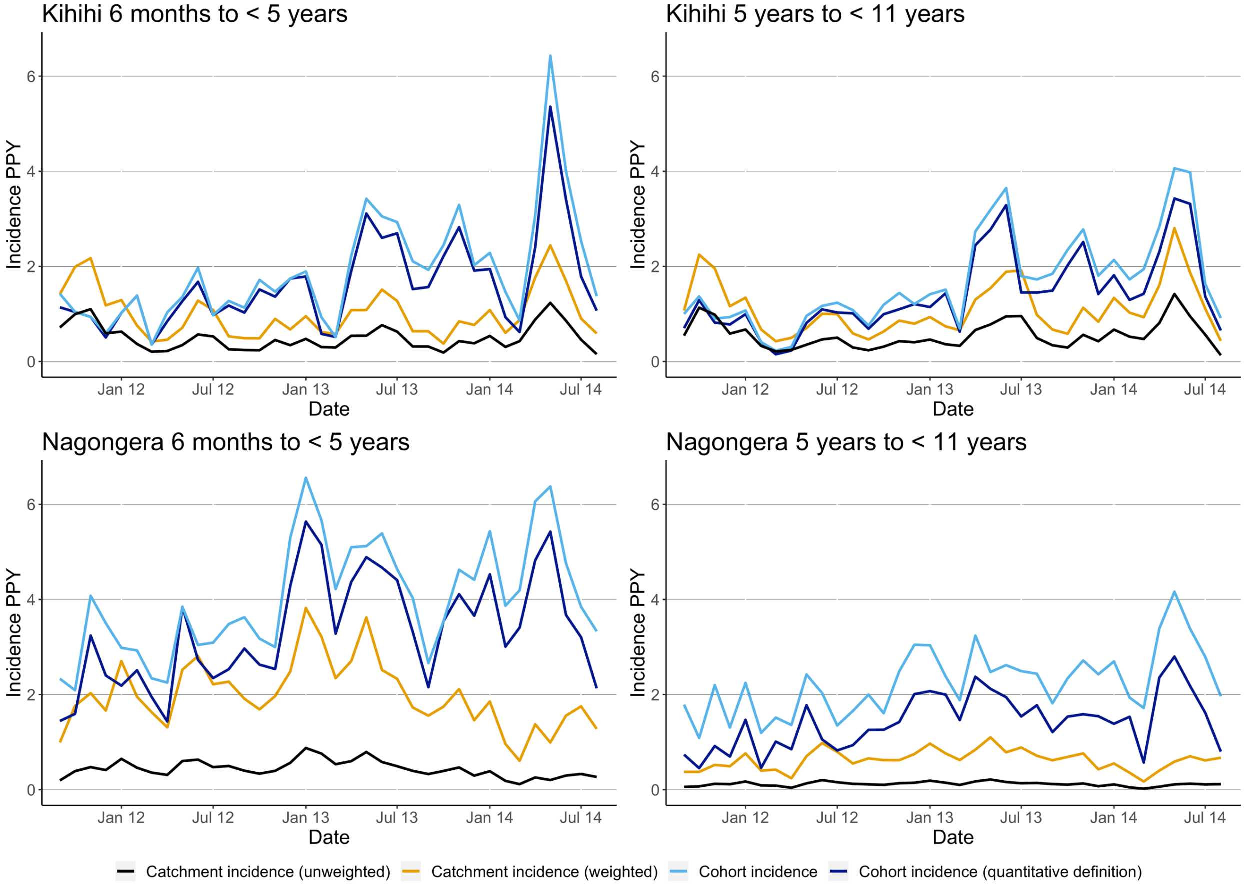

Supplement: Supplementary file 6 — Additional file 6: Incidence of malaria over the 3-year observation period measured in cohorts with additional parasite threshold definition and using health facility-based surveillance. [file 12936_2020_3514_MOESM6_ESM.pdf]
